# Mapping GLP-1 receptor agonist research across the MASLD–MASH–HCC continuum: a bibliometric and translational analysis

**DOI:** 10.3389/fmed.2026.1913929

**Published:** 2026-08-14

**Authors:** Yannan Xie, Xusheng Zhang, Bendong Chen

**Affiliations:** 1 Department of Hepatobiliary Surgery, People’s Hospital of Ningxia Hui Autonomous Region, Yinchuan, China; 2 Ningxia Medical University, Yinchuan, China

**Keywords:** bibliometrics, glucagon-like peptide-1 receptor agonists, hepatocellular carcinoma, incretin-based therapy, metabolic dysfunction-associated steatohepatitis, metabolic dysfunction-associated steatotic liver disease

## Abstract

**Background:**

Metabolic dysfunction increasingly shapes the burden of hepatocellular carcinoma (HCC) by linking obesity, type 2 diabetes, insulin resistance, and metabolic dysfunction-associated steatotic liver disease (MASLD) to steatohepatitis, fibrosis, cirrhosis, and HCC. GLP-1 receptor agonists (GLP-1RAs) target several upstream metabolic drivers, but the structure and evolution of research connecting GLP-1RAs with the MASLD–MASH–HCC continuum remain unclear.

**Methods:**

We searched Web of Science Core Collection, PubMed, and Scopus for English-language articles and reviews published from January 1, 2000, to May 25, 2026. A GLP-1RA or related incretin concept was mandatory for inclusion, together with relevance to HCC and metabolic liver disease. After removal of 450 duplicates and relevance screening, 534 publications were analyzed for publication trends, collaboration networks, journals, authors, and keyword evolution. Bibliometric themes were subsequently interpreted through an expert synthesis of independent mechanistic and clinical evidence.

**Results:**

Research output accelerated after 2020; the United States remained the leading contributor, while China showed rapid recent growth. Keyword evolution shifted from glucose lowering, obesity, and insulin resistance toward MASLD/MASH, liver fibrosis, HCC risk, drug therapy, and clinical outcomes. These bibliometric patterns informed—but did not independently establish—a four-domain interpretive framework comprising systemic metabolic remodeling, intrahepatic lipotoxicity relief, inflammation–fibrosis regulation, and HCC risk and premalignant microenvironment modulation, with clinical translation considered across all four domains.

**Conclusion:**

Research linking GLP-1RAs to the MASLD–MASH–HCC continuum is expanding rapidly. The available evidence suggests that GLP-1RAs are best investigated as upstream disease-modifying and risk-reducing agents in metabolically high-risk populations rather than as established direct anti-HCC therapies. Prospective studies with stage-specific liver and cancer outcomes are required to test this translational hypothesis.

## 1 Introduction

Hepatocellular carcinoma (HCC) remains one of the most lethal malignancies worldwide, and its etiological landscape is changing (1, 2). Although viral hepatitis and alcohol-related liver disease continue to account for a substantial proportion of cases, metabolic dysfunction has emerged as an increasingly important contributor to the global HCC burden (2, 3). Obesity and type 2 diabetes mellitus (T2DM) are no longer merely background comorbidities; both have been associated with increased risks of liver cancer and HCC in epidemiological studies and meta-analyses (4, 5). Consistent with this shift, Global Burden of Disease estimates indicate a measurable liver cancer burden attributable to high body-mass index and high fasting plasma glucose, underscoring the public health relevance of modifiable metabolic risks (3). These GBD-based trends are summarized as contextual evidence in Supplementary Figure S1.

The terminology and clinical framing of metabolic liver disease have evolved. Non-alcoholic fatty liver disease (NAFLD) and non-alcoholic steatohepatitis (NASH) are retained when referring to historical studies, whereas metabolic dysfunction-associated steatotic liver disease (MASLD) and metabolic dysfunction-associated steatohepatitis (MASH) are used for contemporary disease framing. The MAFLD construct is reported only when required by the source literature or search strategy. Updated European guidance emphasizes case finding, non-invasive fibrosis risk stratification, lifestyle and cardiometabolic management, and individualized longitudinal care (6–10). Together, these concepts support a dynamic continuum linking systemic metabolic stress, steatosis, lipotoxicity, inflammation, fibrosis, cirrhosis, and HCC (11–15).

GLP-1 receptor agonists (GLP-1RAs) have rapidly evolved from glucose-lowering agents into broader metabolic therapies. Their effects on weight reduction, glycemic control, insulin resistance, cardiovascular risk, and steatohepatitis make them directly relevant to early drivers of metabolic liver disease (16–18). Clinical studies of liraglutide, semaglutide, and tirzepatide have further strengthened interest in incretin-based therapies for steatotic liver disease and MASH (17–20). However, the relationship between GLP-1RAs and HCC is not straightforward. These agents should not be regarded simply as anti-cancer drugs; the more relevant question is whether they can modify the metabolic and hepatic conditions from which an increasing proportion of HCC arises.

The literature connecting GLP-1RAs, MASLD/MASH, and HCC is expanding but remains fragmented across endocrinology, hepatology, oncology, and pharmacology (14, 21). Unlike a narrative review, bibliometric analysis can quantify temporal growth, collaboration structure, influential sources, and thematic evolution across a large corpus. We therefore mapped this research landscape and used the resulting themes to guide an expert interpretive synthesis of four interconnected mechanistic domains: systemic metabolic remodeling, intrahepatic lipotoxicity relief, inflammation–fibrosis regulation, and HCC risk and premalignant microenvironment modulation. The biological framework is an evidence-informed interpretation of the bibliometric results, not a direct output of network algorithms.

## 2 Materials and methods

### 2.1 Data collection and search strategy

Global burden data were obtained from the IHME GBD Results Tool and used only as contextual evidence; they were not merged with the bibliometric corpus. Literature searches were completed on May 25, 2026, in Web of Science Core Collection, PubMed, and Scopus for records published from January 1, 2000, to May 25, 2026. Eligible publication types were English-language articles and reviews. The search combined a mandatory GLP-1RA/incretin concept with HCC/liver cancer and metabolic liver disease concepts, including current and legacy terminology. Database-specific field tags and controlled vocabulary were adapted to each platform.

The strategy comprised three mandatory Boolean blocks covering GLP-1RAs and related incretin therapies, HCC/liver cancer, and metabolic or liver-related conditions. Complete database-specific search strategies, including field tags, Boolean operators, filters, the execution date, and retrieved record counts, are provided in Supplementary Methods S1.

### 2.2 Literature screening and data processing

**Figure 1:**
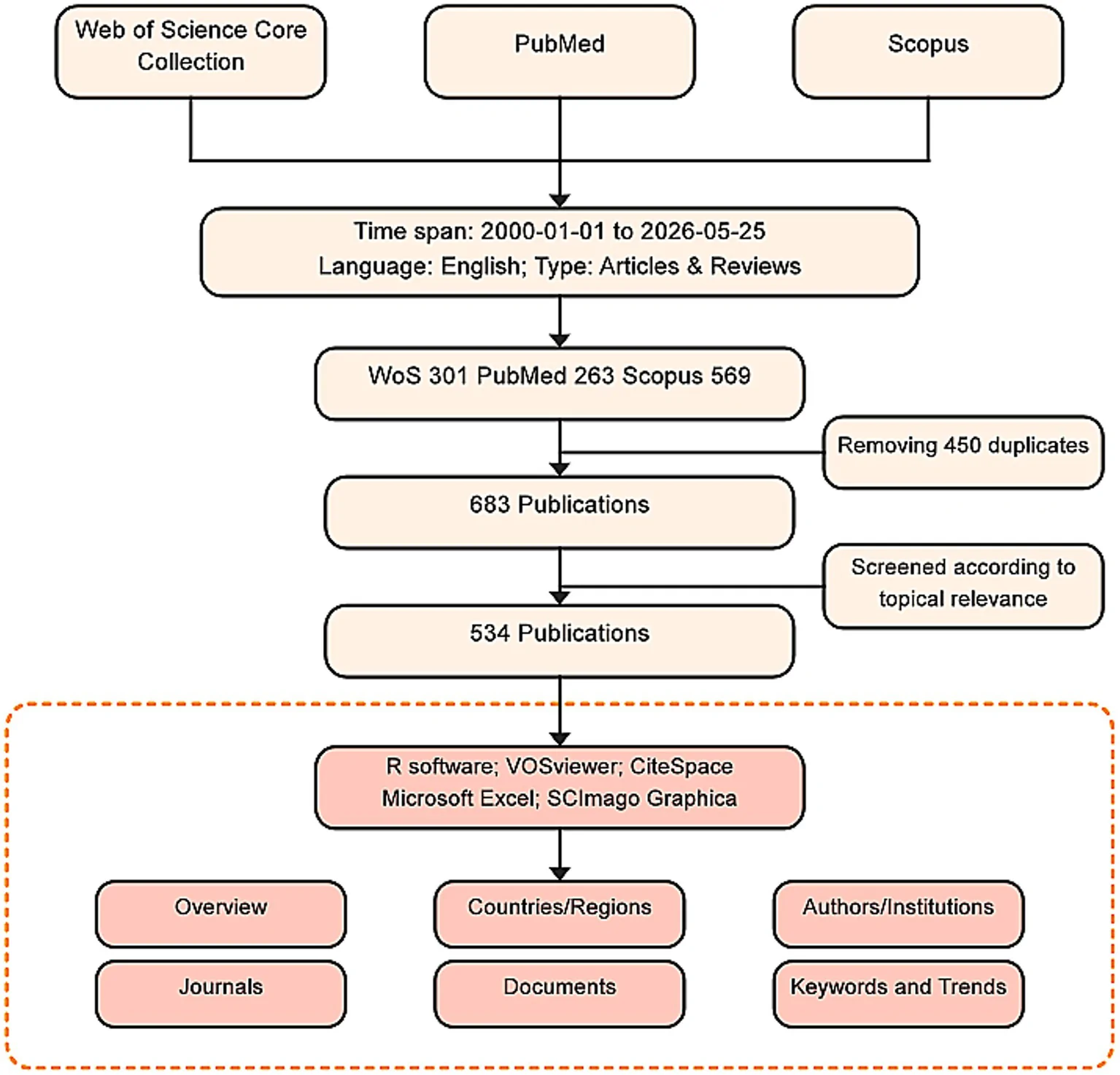
Study selection and bibliometric analysis workflow.

Searches yielded 1,133 records (301 from Web of Science, 263 from PubMed, and 569 from Scopus). Following metadata harmonization and Zotero-assisted deduplication, 450 duplicates were removed, leaving 683 unique records. Two reviewers (YX and XZ) independently screened titles and abstracts against prespecified eligibility criteria; disagreements were resolved by discussion and, when required, adjudicated by BC. Inter-reviewer agreement was not formally quantified. Among the 683 records, 119 met the broad search criteria but lacked a sufficiently direct analytical connection among GLP-1RA exposure, metabolic liver disease, and HCC and were retained only in the expanded dataset; two were retained solely as mechanistic background because HCC was not an explicit focus. Twenty-seven records failed at least one eligibility criterion (language, document type, or date range), and one lacked sufficient relevance to metabolic HCC. Excluding these 149 records from the primary analysis yielded 534 publications in the Main_Core dataset (Figure 1).

### 2.3 Bibliometric analysis

Analyses were performed using R version 4.5.2 and bibliometrix version 5.3.0 through Biblioshiny version 1.13.0 (22), VOSviewer version 1.6.20 (23), and CiteSpace version 6.4. R1 (24). VOSviewer networks used full counting, association-strength normalization, and default clustering settings (resolution 1.00, minimum cluster size 1, with small-cluster merging enabled). Minimum thresholds were one publication for countries (53/53), two publications for institutions (150/841), three publications for authors (78/2,545), three publications for journals (42/266), and 55 occurrences for all keywords (90/6,206). All keywords comprised author-supplied keywords and database-assigned index keywords. To preserve historical terminology and indexing heterogeneity, no manual keyword deletion or synonym merging was performed; NAFLD/NASH, MAFLD, and MASLD/MASH were retained as separate terms. CiteSpace used one-year time slices, Top N = 50 per slice, and minimum-spanning-tree pruning; other burst and clustering parameters remained at their default values. Institutional and author name variants were standardized conservatively. No formal sensitivity analysis was performed.

## 3 Results

### 3.1 Overall trend analysis

**Figure 2:**
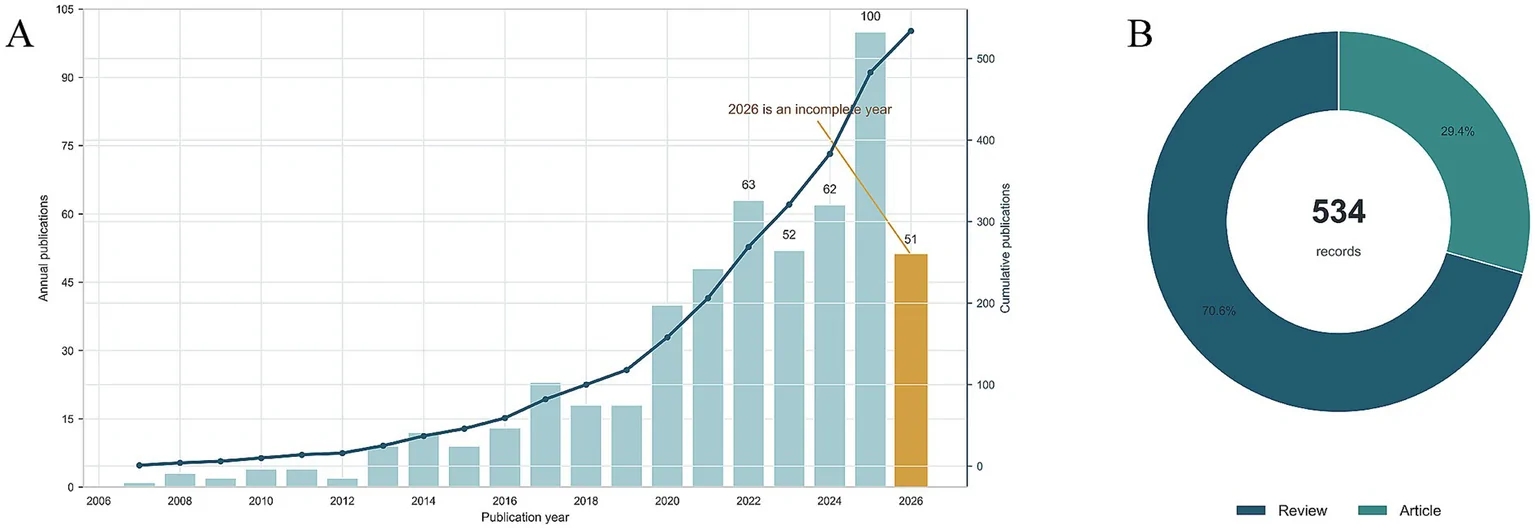
Overall publication trends: **(A)** Annual and cumulative publications; **(B)** document-type distribution.

A total of 534 publications were included. Output remained low from 2007 to 2016, increased after 2017, and accelerated from 40 publications in 2020 to 100 in 2025, corresponding to a 2020–2025 compound annual growth rate of 20.1%. The 51 publications indexed by May 25, 2026, represent an incomplete year and should not be interpreted as a decline (Figure 2A). Reviews accounted for 70.6% of the corpus and original articles for 29.4% (Figure 2B). The field has accumulated extensive synthesis and mechanistic discussion, while original clinical and experimental evidence remains comparatively limited.

### 3.2 Country/region analysis

**Figure 3:**
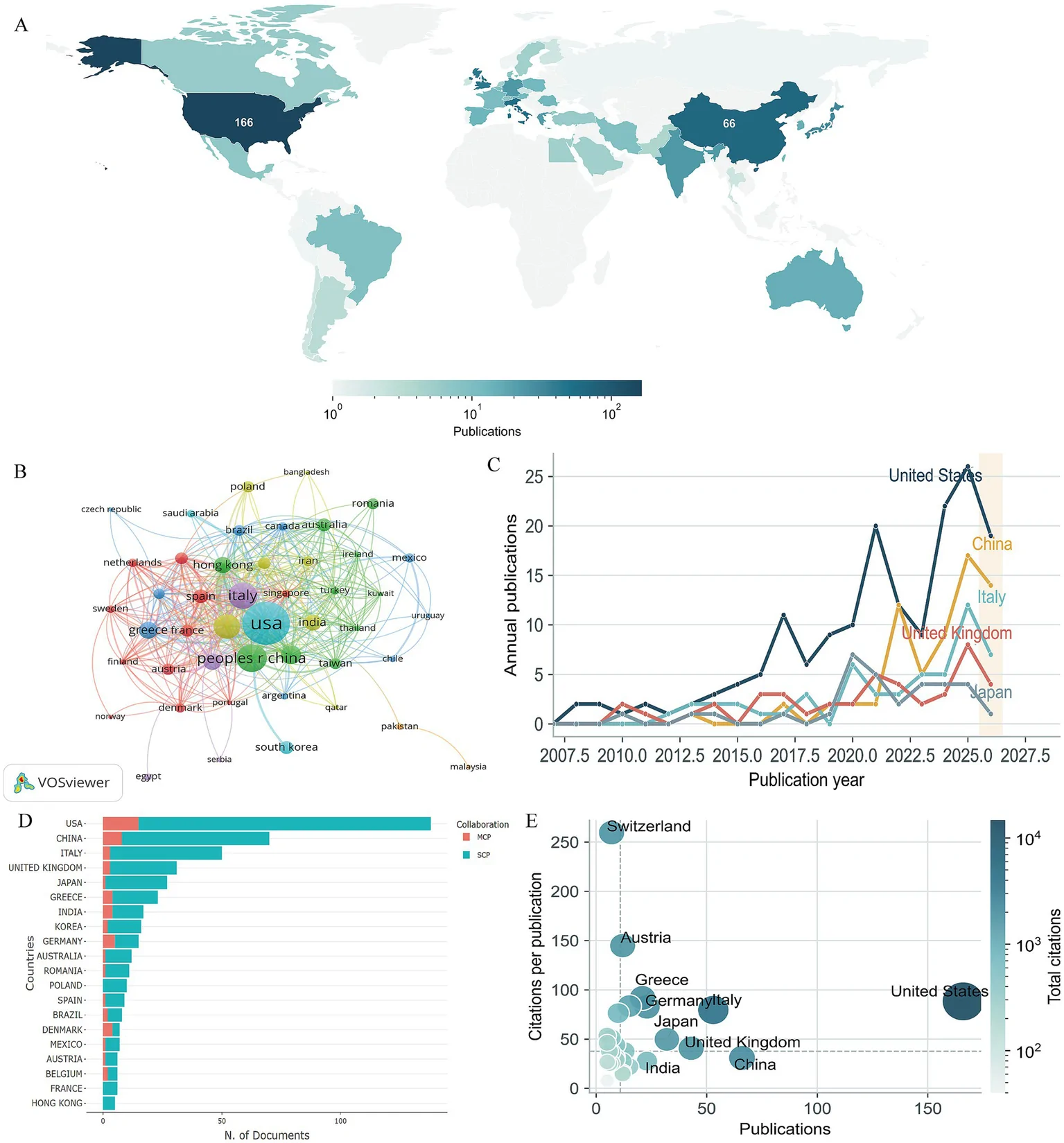
Country/region analysis: **(A)** Global publication distribution based on full counting of all author affiliations; **(B)** collaboration network; **(C)** annual output of leading countries; **(D)** corresponding-author country output divided into single-country and multiple-country publications; **(E)** publication output and citation impact.

The full-counting affiliation analysis included all 53 identified countries/regions, using a minimum threshold of one publication. The United States ranked first with 166 publications, followed by China with 66 (Figure 3A), and occupied the central position in the collaboration network (Figure 3B). Output increased markedly after 2020, with the United States remaining dominant and China showing rapid recent growth (Figure 3C). In the separate corresponding-author country analysis, single-country publications predominated among the most productive countries, indicating limited international coauthorship (Figure 3D; Supplementary Table S1). The United States combined high productivity with substantial citation impact, whereas several lower-output European countries, particularly Switzerland, achieved higher citations per publication (Figure 3E).

### 3.3 Institutional analysis

**Figure 4:**
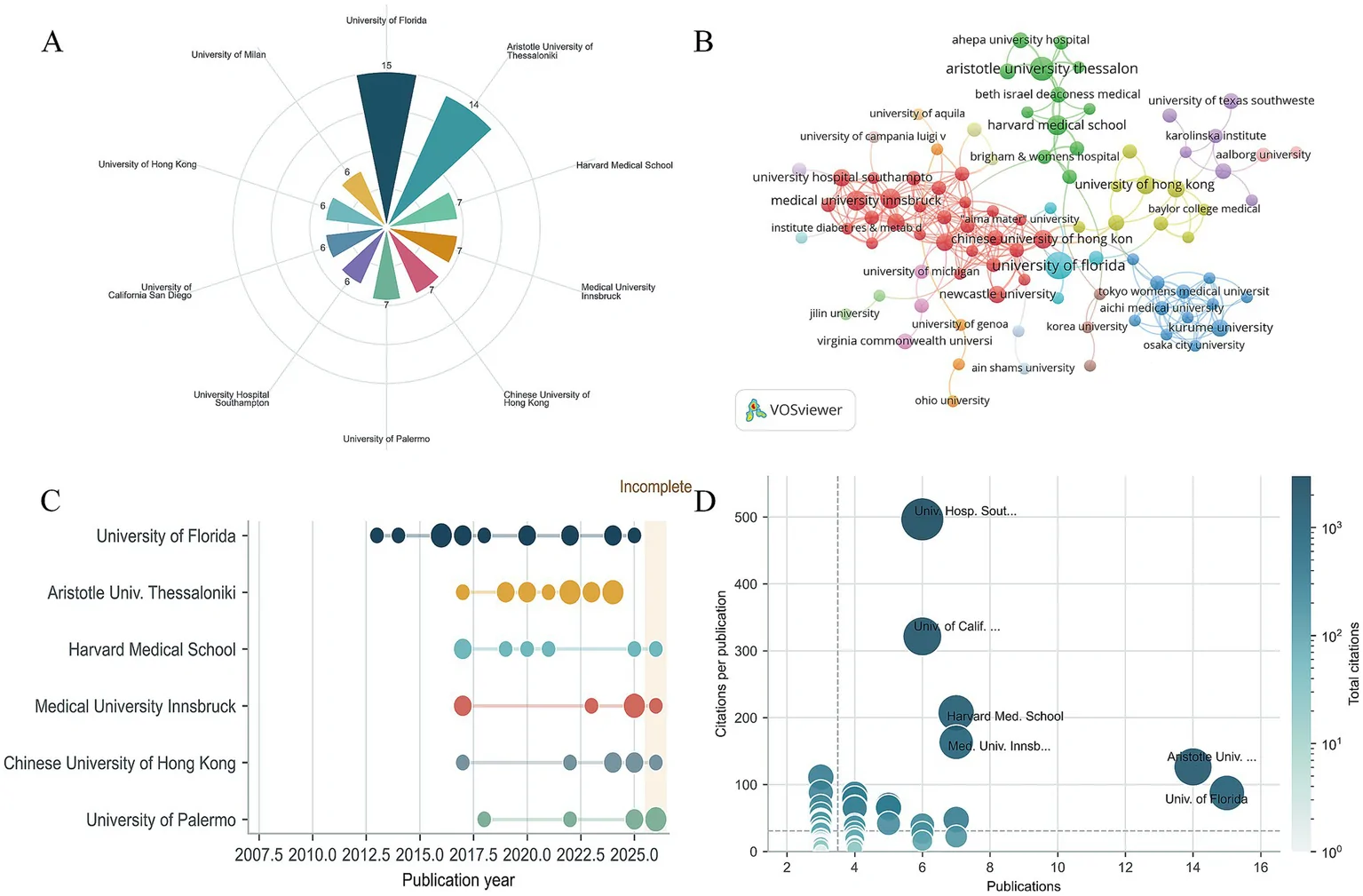
Institutional analysis: **(A)** Leading institutions; **(B)** collaboration network; **(C)** annual publication trends; **(D)** publication output and citation impact.

Among the 841 institutions identified, 150 met the minimum threshold of at least two publications and were included in the network analysis. The University of Florida and Aristotle University of Thessaloniki ranked highest in publication output, followed by Harvard Medical School, Medical University Innsbruck, and the Chinese University of Hong Kong (Figure 4A). Several productive institutions had established cross-regional links, although the overall network remained dispersed rather than forming a highly integrated global collaboration system (Figure 4B). The University of Florida entered the field relatively early and maintained continuous output, whereas several European and Asian institutions became more active in recent years; the incomplete 2026 data should be interpreted cautiously (Figure 4C). Productivity was not fully aligned with mean citation impact: high-output institutions contributed sustained activity, while some lower-output institutions produced more highly cited work (Figure 4D).

### 3.4 Author analysis

**Figure 5:**
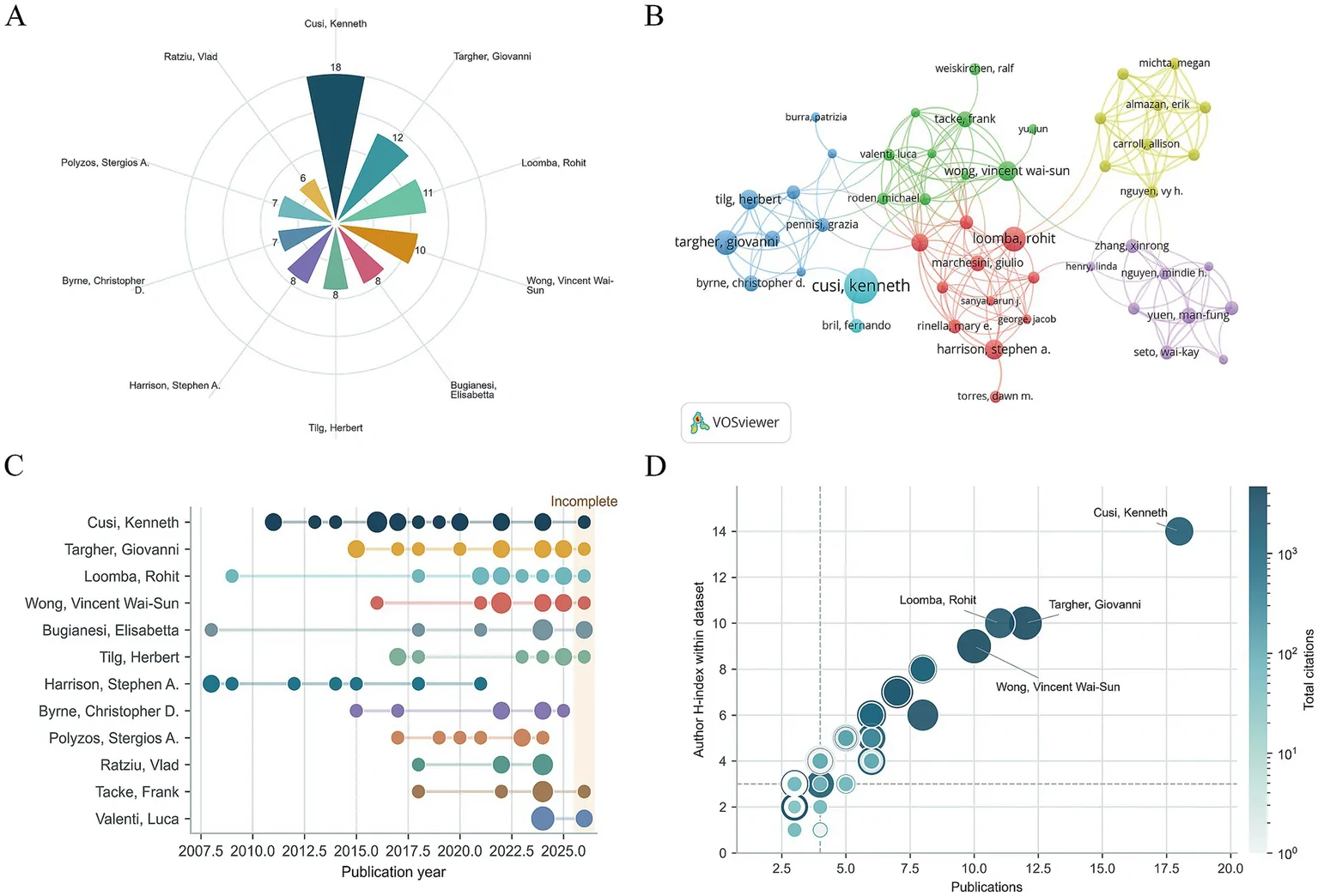
Author analysis: **(A)** Leading authors; **(B)** collaboration network; **(C)** annual publication trends; **(D)** publication output and academic impact.

Among the 2,545 authors identified, 78 met the minimum threshold of at least three publications and were included in the network analysis. Kenneth Cusi had the highest publication output, followed by Giovanni Targher, Rohit Loomba, Vincent Wong, and other leading contributors (Figure 5A). The collaboration network showed a clear team-based structure (Figure 5B). Some core authors entered the field early and maintained continuous output, while new contributors became increasingly active in recent years (Figure 5C). Kenneth Cusi also ranked highly in h-index and total citations, indicating substantial academic influence (Figure 5D; Supplementary Table S2).

### 3.5 Journal analysis

**Figure 6:**
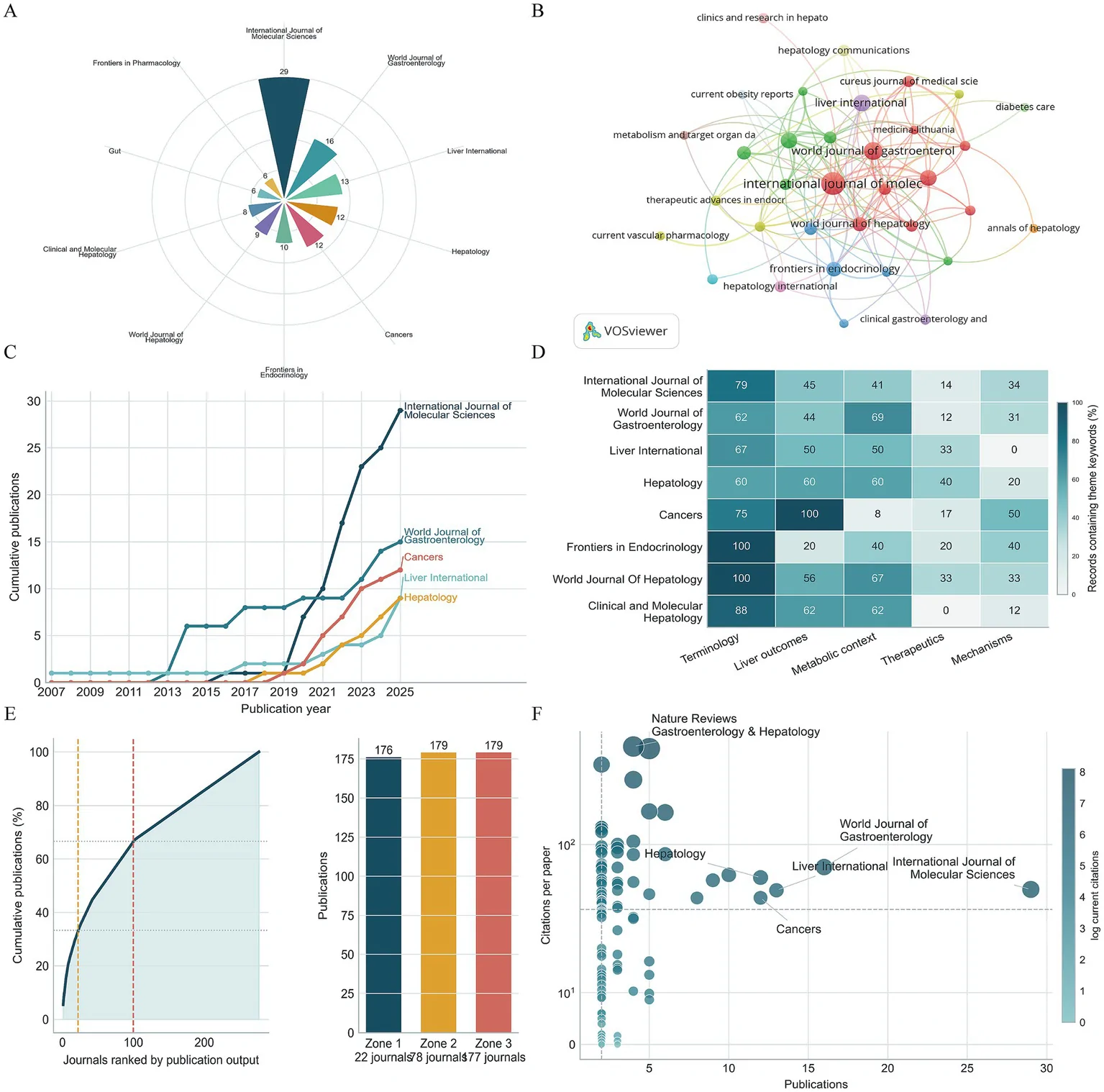
Journal analysis **(A)** leading journals; **(B)** journal association network; **(C)** cumulative publication trends; **(D)** thematic profile heatmap; **(E)** Bradford zones; **(F)** publication output and citation impact.

Of the 266 journals identified, 42 published at least three relevant articles and were included in the analysis. The International Journal of Molecular Sciences had the highest publication output, followed by the World Journal of Gastroenterology, Liver International, and Hepatology (Figure 6A). Hepatology, gastroenterology, endocrinology, and molecular medicine journals formed interconnected publication clusters, reflecting the interdisciplinary nature of the field (Figure 6B). Growth in major journals occurred mainly after 2020 and was most pronounced in the International Journal of Molecular Sciences (Figure 6C). The thematic heatmap suggested that hepatology journals emphasized liver outcomes and metabolic background, molecular and oncology journals emphasized mechanisms, and gastroenterology journals emphasized therapeutic translation (Figure 6D). Bradford analysis showed that 22 Zone 1 journals contributed 176 publications, confirming a core-journal concentration (Figure 6E). High publication volume did not necessarily correspond to the greatest time-adjusted citation impact (Figure 6F; Supplementary Table S3).

### 3.6 Representative publications

All 534 publications collectively contributed to the knowledge structure identified in this study. Because citation indicators are not directly comparable across Web of Science, Scopus, and PubMed, we did not construct a pooled citation ranking. Instead, representative publications were selected from the Main_Core corpus according to their direct relevance to GLP-1RAs and liver-specific or HCC outcomes, together with their complementary clinical, experimental, and evidence-synthesis contributions.

At the population level, the most direct HCC evidence was provided by Wang et al., who analyzed 1,890,020 patients with T2DM and found that GLP-1RA use was associated with a lower risk of incident HCC than insulin or sulfonylureas, together with lower risks of hepatic decompensation across several antidiabetic comparators (21). Complementary cohort studies examined progression through clinically relevant pre-HCC stages. Engström et al. reported fewer serious liver events among GLP-1RA initiators than among DPP-4 inhibitor initiators (HR, 0.85; 95% CI, 0.75–0.97). This association was driven primarily by fewer cirrhosis events, whereas the HCC-specific estimate was not significant (25). Kanwal et al. further found that GLP-1RA use was associated with a 14% lower risk of progression to cirrhosis among patients with MASLD and diabetes without pre-existing cirrhosis, whereas the association was not evident after cirrhosis had developed (26).

Experimental studies provided early biological support for HCC-related effects. Exenatide reduced tumor multiplicity in obesity-associated HCC models and suppressed EGFR–STAT3 signaling and downstream genes involved in proliferation and apoptosis resistance (27). In a diabetic NASH–HCC mouse model, liraglutide improved steatosis, inflammation, and hepatocyte ballooning and suppressed HCC development during the study period (28).

Evidence syntheses nevertheless highlighted substantial uncertainty. One meta-analysis found a lower HCC risk with GLP-1RAs than with insulin or no GLP-1RA exposure, but no significant difference compared with metformin or DPP-4 inhibitors (29). A separate meta-analysis of 90 randomized trials did not detect a significant association between GLP-1RA use and hepatic cancer risk (RR, 0.79; 95% CI, 0.51–1.21) (30). Together, these studies document the evolution of the evidence base from experimental hepatocarcinogenesis to pre-HCC liver outcomes and population-level HCC-risk assessment. They support further evaluation of GLP-1RAs as potential upstream modifiers of liver and HCC risk, while direct anti-HCC efficacy and causal prevention remain unestablished.

### 3.7 Keyword co-occurrence analysis

**Figure 7:**
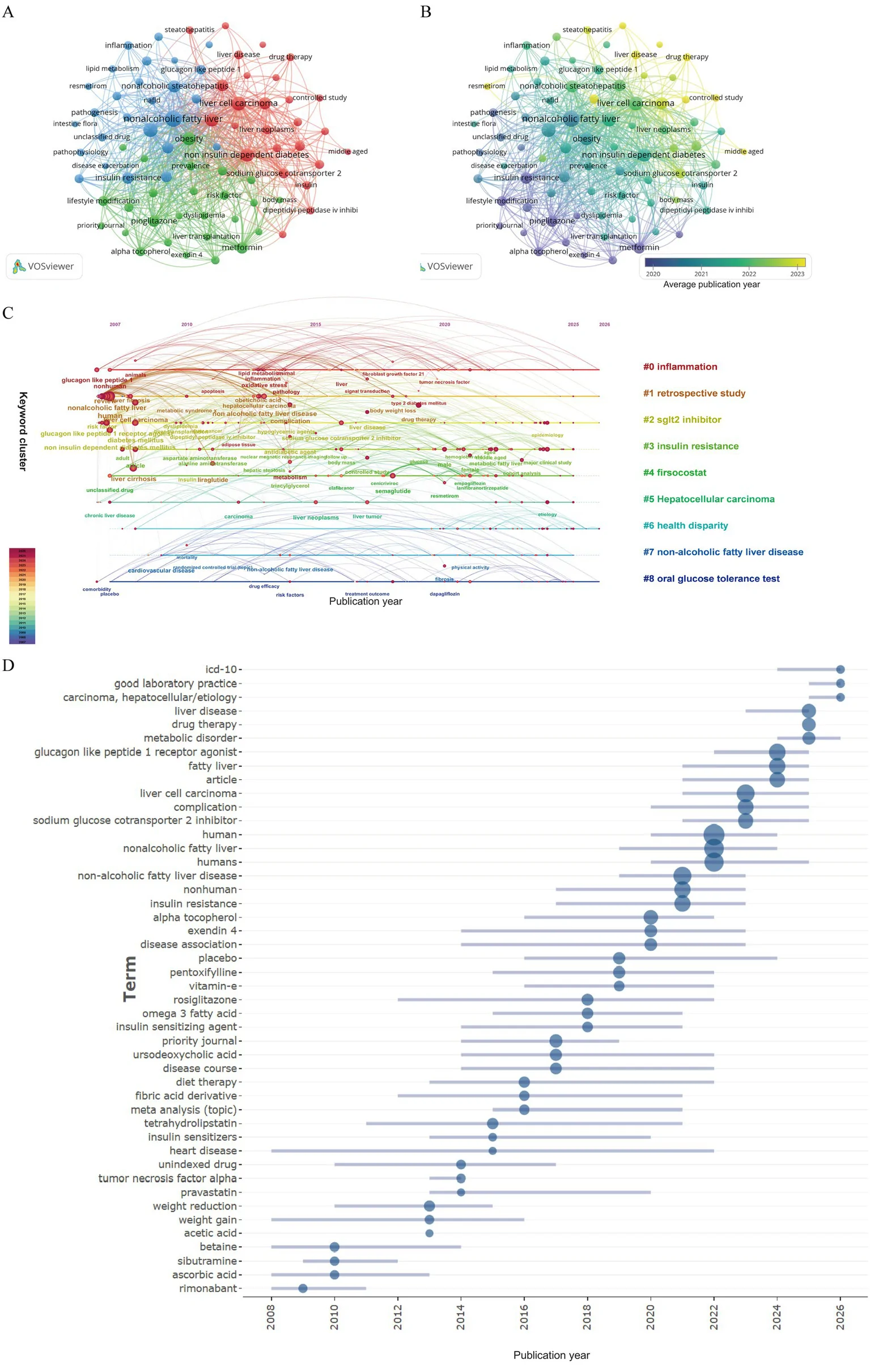
Keyword analysis: **(A)** Co-occurrence network; **(B)** overlay visualization; **(C)** cluster timeline; **(D)** burst terms.

Analysis of the unmerged all-keyword set showed several closely connected thematic clusters centered on diabetes, obesity, historical and current steatotic liver disease terminology, HCC, drug therapy, and inflammatory mechanisms (Figure 7A). Overlay visualization indicated that earlier studies emphasized glucose metabolism, insulin resistance, and weight-related topics, whereas recent hotspots increasingly included MASLD/MASH, SGLT2 inhibitors, liver cancer, and clinical outcomes (Figure 7B). Timeline analysis identified inflammation, retrospective study, SGLT2 inhibitor, insulin resistance, hepatocellular carcinoma, and non-alcoholic fatty liver disease as major evolving themes (Figure 7C). Recent burst terms included liver disease, drug therapy, GLP-1 receptor agonist, fatty liver, and liver cell carcinoma, suggesting a shift from traditional metabolic regulation toward liver outcomes, tumor risk, and clinical translation (Figure 7D). Because synonyms and legacy disease labels were intentionally not merged, semantically related terms may appear as separate nodes.

## 4 Discussion

This study mapped 534 publications connecting GLP-1RAs with metabolic liver disease and HCC. Publication growth accelerated after 2020, international collaboration remained dispersed, and keyword evolution moved from obesity, insulin resistance, metformin, and metabolic syndrome toward GLP-1 receptor, semaglutide, MASH, fibrosis, HCC, and drug therapy. These network patterns identify dominant and emerging research themes but do not establish biological causality. We therefore used the bibliometric findings to guide an expert synthesis of independent mechanistic and clinical evidence. The resulting framework comprises four mechanistic domains—systemic metabolic remodeling, intrahepatic lipotoxicity relief, inflammation–fibrosis regulation, and HCC risk/premalignant microenvironment modulation—with clinical translation treated as a cross-domain component rather than a fifth biological stage.

### 4.1 Domain 1—systemic metabolic remodeling: reducing upstream pro-carcinogenic pressure

At the start of the MASLD–MASH–HCC continuum, GLP-1RAs are best viewed as upstream metabolic disease modifiers rather than direct antitumor agents. Obesity and T2DM increase HCC risk (4, 5), while contemporary MASLD guidance frames steatosis, metabolic stress, fibrosis risk, and extrahepatic cardiometabolic disease as an integrated, dynamic clinical problem (6–10, 13, 15). The relevant question is therefore whether intervention before irreversible liver injury can reduce the metabolic conditions that permit carcinogenesis.

GLP-1RAs improve glucose homeostasis through pancreatic beta-cell cAMP–PKA/Epac2 signaling and reduce energy intake through central POMC/CART and NPY/AgRP pathways (31–34). The clinically substantial weight loss achieved with semaglutide and tirzepatide (16, 35) can decrease adipose lipolysis, portal free-fatty-acid delivery, insulin resistance, and hepatic metabolic load. These effects are more directly supported than any putative direct action on HCC cells.

Improved insulin sensitivity may also reduce compensatory hyperinsulinemia and persistent insulin/IGF-1 activation of PI3K–AKT–mTOR and RAS–MAPK signaling (36, 37). In parallel, reduced adipose macrophage infiltration and correction of adipokine imbalance may attenuate systemic TNF-alpha, IL-6, IL-1beta, MCP-1, JAK/STAT, and NF-kappaB signaling (38–40). Modulation of the gut–liver axis and LPS–TLR4–MyD88–NF-kappaB signaling remains plausible but less firmly established in humans (41). Domain 1 positions GLP-1RAs as modifiers of the host metabolic environment. The observed association with lower HCC and hepatic decompensation risk in T2DM is encouraging but remains observational (21) and cannot be interpreted as direct antitumor efficacy.

### 4.2 Domain 2—intrahepatic lipotoxicity relief: interrupting active MASH-related hepatocyte injury

In MASLD/MASH, the therapeutic focus shifts from systemic metabolic unloading to the hepatic consequences of excess lipid delivery and synthesis. Increased adipose free-fatty-acid influx, *de novo* lipogenesis, impaired oxidation, and altered VLDL export expose hepatocytes to sustained metabolic stress (42, 43). GLP-1RAs may interrupt this transition chiefly through weight loss and improved insulin resistance rather than through a proven direct hepatocyte receptor effect.

Reduced portal lipid delivery and lower SREBP-1c, ChREBP, ACC, FASN, and SCD1 drive can decrease hepatic fat accumulation (42, 43). The D-LIFT trial and MRI-based semaglutide study demonstrated reductions in liver fat, although liver stiffness did not consistently improve (44, 45). These findings locate the most reproducible early benefit at the level of steatosis and metabolic activity, before established structural fibrosis.

This distinction matters because diacylglycerols, ceramides, free cholesterol, saturated fatty acids, and oxidized lipids—not triglyceride storage alone—promote PKCepsilon activation, impaired AKT signaling, mitochondrial ROS, endoplasmic reticulum stress, and hepatocyte death–regeneration cycles (42, 46). Long non-coding RNAs can further integrate *de novo* lipogenesis, beta-oxidation, oxidative stress, mTOR signaling, apoptosis, and fibrogenesis across hepatocytes, Kupffer cells, and stellate cells (47). Recent work on the hepatic PTPRD–STAT3 axis also illustrates how disturbed metabolic signaling can link lipid homeostasis, unfolded-protein responses, and MASH biology (48).

### 4.3 Domain 3—inflammation–fibrosis regulation: delaying a tumor-promoting hepatic niche

Fibrosis stage is the strongest pathological predictor of liver-related events, HCC risk, and mortality in MASLD (49–54). Importantly, normal aminotransferases do not exclude clinically meaningful fibrosis; patients with fibrosis and persistently normal ALT may have a less favorable response to lifestyle treatment and may require greater weight and liver-fat reduction to improve stiffness (55). Risk stratification should therefore rely on validated non-invasive fibrosis pathways rather than ALT alone (7, 8, 10, 54).

Persistent lipotoxicity and oxidative stress activate Kupffer cells and monocyte-derived macrophages, increasing TNF-alpha, IL-1beta, IL-6, CCL2, JNK, NF-kappaB, and NLRP3 signaling (49, 52). By reducing hepatic lipid influx, mitochondrial stress, and hepatocyte injury, GLP-1RAs may lower the inflammatory signals that sustain fibrogenesis. Preclinical GLP-1 pathway studies support reductions in inflammatory infiltration and fibrosis-related expression (56), but direct human hepatic immunomodulation remains incompletely established.

TGF-beta, PDGF, ROS, bile acids, and macrophage-derived signals activate hepatic stellate cells and induce alpha-SMA, COL1A1, TIMP1, collagen deposition, and matrix remodeling (50, 51). Because human cellular studies do not demonstrate consistent direct GLP-1/GIP agonist actions in hepatocytes or stellate cells (57), the defensible interpretation is indirect reduction of fibrogenic drive. Trial data suggest the greatest opportunity before fixed cirrhosis, particularly during active MASH or F2–F3 fibrosis (17–20, 54, 58). Domain 3 links GLP-1RA-mediated metabolic unloading to lower inflammatory and stellate-cell stimulation. This is a disease-modifying hypothesis for slowing fibrotic niche formation, not a claim that GLP-1RAs directly reverse advanced fibrosis.

### 4.4 Domain 4—HCC risk and premalignant microenvironment modulation: from treatment claims to precision prevention

At the distal end of the continuum, GLP-1RAs should be evaluated for HCC risk modification before cancer develops, not presented as established therapy for existing HCC. MASLD/MASH-associated carcinogenesis reflects the cumulative interaction of metabolic injury, inflammation, fibrosis, genetics, and immune dysfunction (15, 59, 60). Accordingly, the central translational hypothesis is that improving the metabolic–inflammatory–fibrotic background may lower long-term carcinogenic pressure.

Hyperinsulinemia and IGF-1 signaling activate PI3K–AKT–mTOR and RAS–MAPK pathways, while IL-6/JAK/STAT3, TNF-alpha/NF-kappaB, and oxidative stress promote repeated injury, abnormal regeneration, and genomic instability (36, 60). The PTPRD–STAT3 metabolic axis provides a recent mechanistic example linking disturbed glucose/lipid homeostasis with MASH biology (48). GLP-1RAs may reduce these inputs through weight loss, insulin sensitization, and lower adipose and hepatic inflammation, but such effects remain upstream and indirect.

MASH-related HCC also exhibits an etiology-specific immune context. Dysfunctional CD8 + PD1 + T-cell activation can impair antitumor surveillance and modify immunotherapy response (61), and recent syntheses emphasize that HCC etiology should inform interpretation of the immune microenvironment (62). Reducing lipotoxic and inflammatory injury may improve the premalignant immune background, but GLP-1RAs cannot yet be defined as immunotherapy sensitizers.

Real-world studies associate GLP-1RA use with lower HCC, cirrhosis progression, hepatic decompensation, or serious liver-event risks, especially before established cirrhosis (21, 25, 26). Preclinical studies suggest effects on STAT3-related signaling and NASH-associated hepatocarcinogenesis (27, 28), while meta-analyses have not shown increased liver or gastrointestinal cancer risk (29, 30, 63). These data support prevention-oriented investigation but do not prove causality. Risk modification must also complement, not replace, HCC surveillance; AI-assisted ultrasonography may improve future detection pathways in high-risk metabolic liver disease, although standardization and external validation remain necessary (64).

### 4.5 Cross-domain clinical translation and precision population targeting

Clinical translation spans all four mechanistic domains. Current guidance supports individualized case finding, non-invasive fibrosis staging, weight management, treatment of T2DM and dyslipidemia, and coordinated long-term care (7–10, 13). GLP-1RAs should therefore be embedded within—not substituted for—lifestyle and cardiometabolic management. Statin exposure has been associated with lower progression to high-risk FIB-4 in primary-care MASLD (65), and eHealth interventions can support weight, BMI, and aminotransferase improvement (66); these findings illustrate complementary risk-modification strategies rather than GLP-1RA-specific efficacy.

The most plausible target population comprises patients with obesity or T2DM plus MASLD/MASH and significant steatosis, active disease, or F2–F3 fibrosis. Endpoints should be stage-specific: weight, HbA1c, ALT/AST, insulin resistance, and MRI-PDFF in early MASLD; MASH resolution and absence of fibrosis progression in active MASH; and histological or validated non-invasive fibrosis outcomes in F2–F3 disease. Normal ALT should not be used to exclude fibrosis or to defer risk stratification (55). Existing trials support histological activity in non-cirrhotic disease but not reliable reversal of established cirrhosis (17–20, 58).

To test HCC prevention, future studies must progress from short-term metabolic endpoints to cirrhosis, decompensation, HCC incidence, recurrence, and liver-related mortality. Because conventional trials would require large samples and long follow-up, pragmatic trials and target-trial emulation should define time zero, eligibility, active comparators, treatment strategies, follow-up, and outcome ascertainment explicitly (67). Analyses should stratify by BMI, T2DM, MASH activity, FIB-4, liver stiffness, fibrosis stage, sex, and relevant comorbidity, while addressing confounding by indication and healthy-user, immortal-time, and residual biases.

Implementation must also account for gastrointestinal adverse effects, gallbladder and biliary disease risk, sarcopenia, malnutrition, delayed gastric emptying, tolerability, adherence, and cost (68). Nomenclature changes further complicate longitudinal evidence synthesis (6, 7, 9). A staged program—short-term metabolic and histological validation followed by long-term liver and cancer outcomes—offers a realistic path from bibliometric signals and mechanistic plausibility to testable clinical benefit.

### 4.6 Limitations

This study has several limitations. Database coverage, indexing policies, field structures, and citation data differ across Web of Science, PubMed, and Scopus, so omissions and residual misclassification may remain despite harmonization. Restriction to English-language articles and reviews may introduce language and publication-type bias. The 2026 output is incomplete. Citation indicators are sensitive to publication age, database coverage, self-citation, and cumulative-advantage effects and should not be interpreted as direct measures of scientific quality. Network structure depends on threshold selection, counting, normalization, the decision to retain unmerged terminology, and layout algorithms; no formal sensitivity analysis was performed. Terminology changes across NAFLD/NASH, MAFLD, and MASLD/MASH may have reduced retrieval consistency over time. Finally, bibliometric associations and the proposed expert framework cannot establish that GLP-1RAs causally prevent HCC; current support is derived largely from indirect trial endpoints, observational studies, and preclinical models.

## Data Availability

The original contributions presented in the study are included in the article/Supplementary material, further inquiries can be directed to the corresponding author.
