# Supplementary material for: Mapping GLP-1 receptor agonist research across the MASLD–MASH–HCC continuum: a bibliometric and translational analysis
Source: Front Med (Lausanne). 2026 Aug 14;13:1913929. doi: 10.3389/fmed.2026.1913929 (PMC13522774; doi:10.3389/fmed.2026.1913929)
Supplement: Supplementary file 1 [file Data_Sheet_1.DOCX]

# **Supplementary Methods S1. Database-specific search strategies**

The searches were executed on May 25, 2026, for records published from January 1, 2000, to May 25, 2026. The GLP-1RA/incretin, liver cancer/HCC, and metabolic concepts were mandatory Boolean blocks. Interface limits were applied as specified below.

## **Web of Science Core Collection**

TS=(
("GLP-1" OR "GLP1" OR "glucagon-like peptide-1" OR "glucagon like peptide 1"
OR "GLP-1 receptor agonist*" OR "GLP-1RA*" OR "GLP1RA*"
OR semaglutide OR liraglutide OR exenatide OR dulaglutide
OR lixisenatide OR albiglutide OR efpeglenatide OR exendin-4 OR exendin)
AND
("hepatocellular carcinoma" OR HCC OR "liver cancer" OR "hepatic cancer"
OR hepatoma OR "primary liver cancer")
AND
(metabolism OR metabolic OR obesity OR diabetes OR diabetic
OR insulin OR "insulin resistance"
OR inflammation OR inflammatory OR fibrosis OR fibrogenesis
OR steatosis OR "fatty liver"
OR NAFLD OR NASH OR MASLD OR MASH
OR carcinogenesis OR tumorigenesis OR oncogenesis
OR tumor OR tumour OR cancer
OR proliferation OR apoptosis OR autophagy
OR immunity OR immune OR microenvironment)
)

Search field: Topic (TS). Limits: English; Article or Review; publication years 2000-2026; search executed May 25, 2026. Records retrieved: 301.

## **PubMed**

(
"GLP-1"[Title/Abstract]
OR "GLP1"[Title/Abstract]
OR "glucagon-like peptide-1"[Title/Abstract]
OR "glucagon like peptide 1"[Title/Abstract]
OR "GLP-1 receptor agonist"[Title/Abstract]
OR "GLP-1 receptor agonists"[Title/Abstract]
OR "GLP-1RA"[Title/Abstract]
OR "GLP-1RAs"[Title/Abstract]
OR "GLP1RA"[Title/Abstract]
OR "GLP1RAs"[Title/Abstract]
OR incretin*[Title/Abstract]
OR semaglutide[Title/Abstract]
OR liraglutide[Title/Abstract]
OR exenatide[Title/Abstract]
OR dulaglutide[Title/Abstract]
OR lixisenatide[Title/Abstract]
OR albiglutide[Title/Abstract]
OR efpeglenatide[Title/Abstract]
OR "exendin-4"[Title/Abstract]
OR exendin[Title/Abstract]
)
AND
(
"hepatocellular carcinoma"[Title/Abstract]
OR HCC[Title/Abstract]
OR "liver cancer"[Title/Abstract]
OR "hepatic cancer"[Title/Abstract]
OR hepatoma[Title/Abstract]
OR "primary liver cancer"[Title/Abstract]
)
AND
(
metabolism[Title/Abstract]
OR metabolic[Title/Abstract]
OR obesity[Title/Abstract]
OR diabetes[Title/Abstract]
OR diabetic[Title/Abstract]
OR insulin[Title/Abstract]
OR "insulin resistance"[Title/Abstract]
OR inflammation[Title/Abstract]
OR inflammatory[Title/Abstract]
OR fibrosis[Title/Abstract]
OR fibrogenesis[Title/Abstract]
OR steatosis[Title/Abstract]
OR "fatty liver"[Title/Abstract]
OR NAFLD[Title/Abstract]
OR NASH[Title/Abstract]
OR MASLD[Title/Abstract]
OR MASH[Title/Abstract]
OR carcinogenesis[Title/Abstract]
OR tumorigenesis[Title/Abstract]
OR oncogenesis[Title/Abstract]
OR tumor[Title/Abstract]
OR tumour[Title/Abstract]
OR cancer[Title/Abstract]
OR proliferation[Title/Abstract]
OR apoptosis[Title/Abstract]
OR autophagy[Title/Abstract]
OR immunity[Title/Abstract]
OR immune[Title/Abstract]
OR microenvironment[Title/Abstract]
)

Search field: Title/Abstract. Limits: English; Journal Article or Review; publication dates January 1, 2000-May 25, 2026; search executed May 25, 2026. Records retrieved: 263.

## **Scopus**

TITLE-ABS-KEY (
"GLP-1" OR GLP1 OR "glucagon-like peptide-1" OR "glucagon like peptide 1"
OR "GLP-1 receptor agonist" OR "GLP-1 receptor agonists"
OR GLP-1RA OR GLP-1RAs OR GLP1RA OR GLP1RAs
OR incretin*
OR semaglutide OR liraglutide OR exenatide OR dulaglutide
OR lixisenatide OR albiglutide OR efpeglenatide
OR "exendin-4" OR exendin
)
AND
TITLE-ABS-KEY (
"hepatocellular carcinoma" OR HCC OR "liver cancer" OR "hepatic cancer"
OR hepatoma OR "primary liver cancer"
)
AND
TITLE-ABS-KEY (
metabolism OR metabolic OR obesity OR diabetes OR diabetic
OR insulin OR "insulin resistance"
OR inflammation OR inflammatory
OR fibrosis OR fibrogenesis
OR steatosis OR "fatty liver"
OR NAFLD OR NASH OR MASLD OR MASH
OR carcinogenesis OR tumorigenesis OR oncogenesis
OR tumor OR tumour OR cancer
OR proliferation OR apoptosis OR autophagy
OR immunity OR immune OR microenvironment
)

Search field: TITLE-ABS-KEY. Limits: English; Article or Review; publication years 2000-2026; search executed May 25, 2026. Records retrieved: 569.

**Supplementary Fig. S1. Global burden of liver cancer attributable to metabolic risk factors. (A and B) Global trends in age-standardized DALY rates attributable to high body-mass index and high fasting plasma glucose from 1990 to 2021. (C and D) Country-level distributions of the corresponding age-standardized DALY rates in 2021. Shaded areas indicate 95% uncertainty intervals. DALY, disability-adjusted life year.**

**Supplementary Table S1. Corresponding-author countries/regions and international collaboration patterns**

| **Country** | **Articles** | **Articles %** | **SCP** | **MCP** | **MCP %** |
| --- | --- | --- | --- | --- | --- |
| USA | 137 | 25.61 | 122 | 15 | 10.9 |
| CHINA | 70 | 13.1 | 62 | 8 | 11.43 |
| ITALY | 50 | 9.3 | 47 | 3 | 6.0 |
| UNITED KINGDOM | 31 | 5.8 | 28 | 3 | 9.7 |
| JAPAN | 27 | 5.0 | 26 | 1 | 3.7 |
| GREECE | 23 | 4.3 | 19 | 4 | 17.4 |
| INDIA | 18 | 3.3 | 14 | 4 | 22.2 |
| KOREA | 16 | 3.0 | 14 | 2 | 12.5 |
| GERMANY | 15 | 2.8 | 10 | 5 | 33.3 |
| AUSTRALIA | 12 | 2.2 | 11 | 1 | 8.3 |

Note: Country assignment in this table was based on the corresponding author's affiliation in Bibliometrix, so each publication was assigned to one country. These counts therefore differ from the full-counting coauthorship network in Fig. 3A, where one publication may be attributed to multiple countries through all author affiliations. SCP, single-country publication; MCP, multiple-country publication.

**Supplementary Table S2. Top contributing authors ranked by local bibliometric impact**

| **Author** | **h_index** | **g_index** | **m_index** | **TC** | **NP** | **PY_start** |
| --- | --- | --- | --- | --- | --- | --- |
| CUSI K | 14 | 19 | 0.87 | 2079 | 19 | 2011 |
| LOOMBA R | 10 | 11 | 0.55 | 2572 | 11 | 2009 |
| TARGHER G | 10 | 12 | 0.83 | 4121 | 12 | 2015 |
| HARRISON SA | 8 | 8 | 0.42 | 1098 | 8 | 2008 |
| TILG H | 8 | 8 | 0.80 | 2103 | 8 | 2017 |
| BYRNE CD | 7 | 8 | 0.58 | 3687 | 8 | 2015 |
| POLYZOS SA | 7 | 7 | 0.70 | 1651 | 7 | 2017 |
| BUGIANESI E | 6 | 8 | 0.31 | 3134 | 8 | 2008 |
| RATZIU V | 6 | 6 | 0.66 | 2453 | 6 | 2018 |
| YONEDA M | 6 | 6 | 0.60 | 488 | 6 | 2017 |

*Note.* h_index, g_index and m_index were calculated within the included bibliometric corpus. TC, total citations; NP, number of publications; PY_start, first publication year in the corpus.

**Supplementary Table S3. Top contributing journals/sources ranked by local bibliometric impact**

| **Source** | **h_index** | **g_index** | **m_index** | **TC** | **NP** | **PY_start** |
| --- | --- | --- | --- | --- | --- | --- |
| INTERNATIONAL JOURNAL OF MOLECULAR SCIENCES | 18 | 29 | 1.6 | 1191 | 29 | 2016 |
| WORLD JOURNAL OF GASTROENTEROLOGY | 14 | 16 | 1.0 | 1022 | 16 | 2013 |
| CANCERS | 11 | 12 | 1.4 | 418 | 12 | 2019 |
| HEPATOLOGY | 8 | 12 | 0.9 | 623 | 12 | 2018 |
| CLINICAL AND MOLECULAR HEPATOLOGY | 7 | 8 | 0.7 | 278 | 8 | 2017 |
| FRONTIERS IN ENDOCRINOLOGY | 7 | 10 | 0.8 | 547 | 10 | 2018 |
| LIVER INTERNATIONAL | 6 | 13 | 0.3 | 525 | 13 | 2007 |
| FRONTIERS IN PHARMACOLOGY | 5 | 6 | 0.6 | 494 | 6 | 2019 |
| GASTROENTEROLOGY | 5 | 5 | 0.3 | 959 | 5 | 2008 |
| GUT | 5 | 6 | 0.6 | 1127 | 6 | 2019 |

*Note.* h_index, g_index and m_index were calculated within the included bibliometric corpus. TC, total citations; NP, number of publications; PY_start, first publication year of the source in the corpus.
